# Communication, Trust and Dental Anxiety: A Person-Centred Approach for Dental Attendance Behaviours

**DOI:** 10.3390/dj8040118

**Published:** 2020-10-13

**Authors:** Siyang Yuan, Ruth Freeman, Kirsty Hill, Tim Newton, Gerry Humphris

**Affiliations:** 1 School of Dentistry, University of Dundee, Dundee DD1 4HN, UK; r.e.freeman@dundee.ac.uk; 2 School of Dentistry, University of Birmingham, Birmingham B5 7EG, UK; k.b.hill@bham.ac.uk; 3 Dental Institute, King’s College, London SE1 1UL, UK; tim.newton@kcl.ac.uk; 4 School of Medicine, University of St Andrews, St Andrews KY16 9TF, UK; gmh4@st-andrews.ac.uk

**Keywords:** person-centred care, dental anxiety, communication, trust, socio-economic status, shame

## Abstract

Effective communication forges the dentist-patient treatment alliance and is thus essential for providing person-centred care. Social rank theory suggests that shame, trust, communication and anxiety are linked together, they are moderated by socio-economic position. The study is aimed to propose and test an explanatory model to predict dental attendance behaviours using person-centred and socio-economic position factors. A secondary data analysis was conducted on a cross-sectional representative survey of a two-stage cluster sample of adults including England, Wales and Northern Ireland. Data were drawn from structured interview. Path analysis of proposed model was calculated following measurement development and confirmation of reliable constructs. The findings show model fit was good. Dental anxiety was predicted negatively by patient’s trust and positively by reported dentist communication. Patient’s shame was positively associated with dental anxiety, whereas self-reported dental attendance was negatively associated with dental anxiety. Both patient’s trust and dentist’s communication effects were moderated by social class. Manual classes were most sensitive to the reported dentist’s communications. Some evidence for the proposed model was found. The relationships reflected in the model were illuminated further when social class was introduced as moderator and indicated dentists should attend to communication processes carefully across different categories of patients.

## 1. Introduction

Person-centred care has been conceptualised to include all aspects of the interaction of an individual with the health care professional and the health care organisation or system. In essence, person-centred care reflected the interconnectedness between the various domains of an individual’s interactions with the totality of health care provision [1]. Recognising the need to enhance quality of care and patient experience, the National Institute of Health and Care Excellence (NICE) in the United Kingdom, promoted person-centred care as an essential element within National Health Service (NHS) treatment provision. In their clinical guidance, NICE, placed the patient at the centre with the clinician when planning, negotiating and providing health care [2].

Person-centred care may have some bearing upon access when an individual attends for dental care. In her seminal paper on accessing dental care, Cohen proposed a series of accessibility factors: those belonging to the patient (e.g., dental anxiety); those to the dental health professional (e.g., attitude) and those related to public health policy as potential barriers to dental attendance [3]. Later researchers supported Cohen’s proposal and pointed to the role of dental anxiety, costs of care, and perception of need as important accessibility factors [4,5]. However, with regard to person-centred care, the significance of the dentist-patient interaction and the role of dental system drivers had not been fully explored [6]. Thus, within the dentist-patient interaction, it was the form of the interaction adopted by the dentist that could leave the person’s dental fears together with their felt and expressed needs hidden and unexplored or voiced and examined [7]. The business context or system drivers of dental practice could further exacerbate the individual’s dental anxiety together with feelings of shame [8,9]. The consequence is to reduce trust within the treatment alliance leading to lowered patient satisfaction and a delay in accessing dental care [5]. The role of such person-centred factors together with a lack of effective communication affected the role of social deprivation with regard to increasing or reducing access to regular dental attendance [4,5,10].

**Figure 1:**
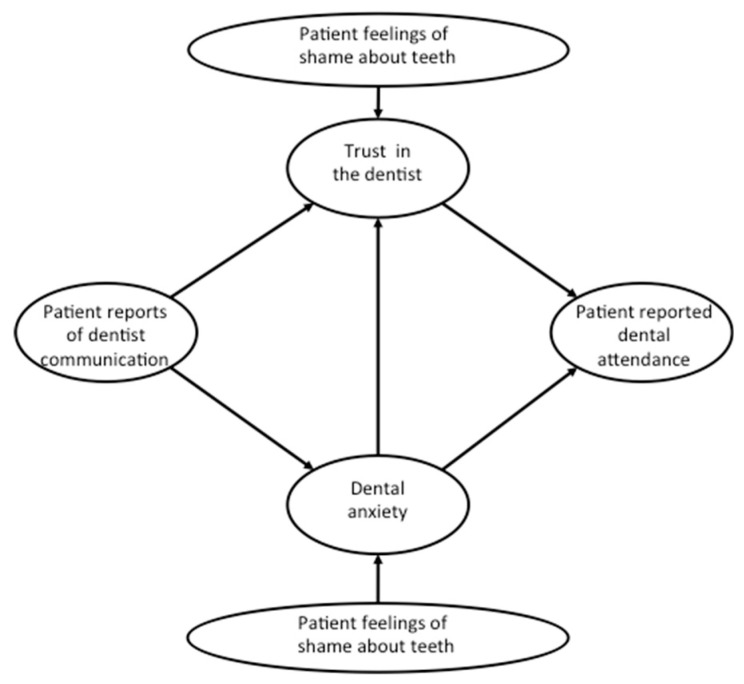
Proposed model between shame, trust, communication and patient dental anxiety.

The importance of person-centred care is now recognised within dentistry as an essential factor in improving dental attendance [7]. Based on Berggren’s vicious circle of dental anxiety, patient’s anxiety was initiated with avoidance of dental attendance, followed by deterioration of oral health and feelings of shame and inferiority, which resulted in further anxiety or fears [8]. In other words, patients’ feelings of shame or inferiority due to poor oral health are part of the vicious circle of dental anxiety. On the other hand, a trusting dentist-patient relationship has a pivotal role in managing patients’ dental anxiety through effective communication [9]. We suggest that shame and trust are significant dimensions of the dentist-patient interaction which have the capacity to influence perceptions of effective communication and increase patient dental anxiety. We propose, therefore, a model to predict dental attendance building on the fundamental features of a person-centred approach. We contend that such variables as [i] dental anxiety, [ii] patient’s trust and [iii] communication [9,10] together with measures of [iv] shame [9,11] are key elements of a person-centred approach. Therefore, these constructs may be configured within a mediator-moderator model that summarises the proposed relationships between dentists’ communication with patient trust and dental anxiety (as mediators) and feelings of shame about teeth (as a moderator) to understand dental attendance (Figure 1). Socio-economic position indicates “the social and economic factors that influence what positions individuals or groups hold within the structure of a society” [12]. As the strength of many of these relationships may be altered by socio-economic position, we proposed to assess the generalisability of the model across major social class groupings [13].

The data from the UK Adult Dental Health Survey (ADHS), presented us with an opportunity to assess the extent of the relationships between these psychological constructs at the population level [14]. The aim of this secondary analysis was, therefore to understand dental attendance using a proposed explanatory mediator-moderator model with dentists’ communication as the principal predictor, patient trust and dental anxiety as mediators and patient shame as a moderator. The three objectives were (i) to estimate the overall model fit (ii) to determine the relationship strength between patients’ trust, dentists’ communication, patient dental anxiety and shame and, (iii) the variation of these relationships across socio-economic positions.

## 2. Materials and Methods

### 2.1. Sample and Procedure

A two-staged weighting approach was applied. Postcode sectors were paired together to alleviate the design effects and enhance the diversity of the population within each PSU. There were 1150 addresses sampled in each of the 10 English Strategic Health Authorities (SHA) and in Wales. In Northern Ireland, 750 addresses were sampled [14]. To obtain similar sample sizes at the SHA level, differential sampling rates were used in SHAs, Wales and Northern Ireland. In addition, a survey weight was applied in order to compensate for these differential rates. We used weighting also to decrease bias that was attributed to non-response. The weighting was also employed to decrease the risk of possible bias attributed to non-response at the interview and examination stages. Although there as limited available information regarding non-responding households, geographic information associated with non-responding households was available from the 2001 Census, which categorises each PSU in terms of significant characteristics including typical household type, social-economic status, ethnicity and so forth. Therefore, it was possible to modify for household non-response given the location a household was in. This was conducted using logistic regression, modelling the probability of response using mid-level output area classification (21 categories). The final stage of the interview weighting ensured that the weights of different age-by-sex groups match the population totals for each SHA, Wales and Northern Ireland for the various age-by-sex groups; this was obtained by integrative calibration [15]. The survey took place between October and December 2009, and January to April 2010. All interviewers were trained and briefed on survey procedure.

### 2.2. Questionnaire and Measures

The ADHS [14] questionnaire included the following measures:

(i) The modified dental anxiety scale was adopted as one of the key independent measures. It consists of five questions to assess dental anxiety which include waiting for treatment, the drill, local anaesthetic injection etc. [16]. It has been shown to have good psychometric properties [17,18], insignificant instrumental effects on respondents and simple to complete [19].
(ii) Two questions from the Oral Health Impact Profile-14 (OHIP-14) [20] were used to assess shame. They asked about the degree of embarrassment and self-consciousness of respondent’s dentition.
(iii) The assessment of self-reported dental attendance was compiled by a weighted index from four questions: (1) regularity, (2) a four category index of past check-up visiting (every six months, every two years, only when in pain or trouble), (3) duration since last visit to the dentist (in months), and (4) the number of check-up visits in the past 5 years.
(iv) Trust was assessed by the four yes/no questions inviting the respondent to indicate whether the dentist listened carefully, the dentist explained reasons for treatment, respondent was treated with respect, and respondent had confidence and trust in dentist.
(v) Communication was assessed by three questions, each with three categories (yes, neutral, no) to show that the respondent ‘got answers that could understand’, was ‘given enough time to discuss’, and was ‘involved as much as wanted’. A summary of factor loadings and internal consistency coefficients derived from the factor analyses to confirm the measurement model are presented in Table 1.
(vi) Socio-demographic status was assessed by a series of questions on educational attainment, income and employment status. This allowed three categories of social class to be constructed which were ‘professional and managerial’, ‘intermediate’ and ‘manual’ [14].

### 2.3. Ethical Issues

The ethics application was submitted to NHS Research Ethics System (NRES) which included the survey in England, Wales and Northern Ireland [14]. The participants were required to fill out their informed consent before participating in the study. The study was conducted in compliance with the Declaration of Helsinki. In addition, the study protocol was approved by the NRES (NRES project number: 09/H0605/50, approved on 5 June 2009).

### 2.4. Statistical Analysis

The measures utilised for model testing were drawn from the above set of survey questions. The question sets were constructed into scales using preliminary exploratory and confirmatory factor analyses. The weighting of each question was determined simultaneously by deriving the factor loadings and the processing of linear functions between the latent variables as estimated by the structural equation procedure in steps described below.

Complete data sets with no missing values on study variables were analysed. The representation of professional respondents was slightly greater than routine manual respondents in this complete version. Two stages of model testing were applied [21]. First the measurement model was tested using the whole sample including those in employment, those non-identified, and those long term unemployed or never worked. The five latent variables were specified by multiple indicators as listed in Table 1. Analyses were run assuming interval level measurement in the indicator variables. Checks were conducted specifying the indicators as ordinal variables using a Bayesian approach and rerun to determine if major differences in the resulting estimates occurred.

On confirmation of the appropriateness of the measurement model, the second analysis stage was conducted consisting of running the structural equation models (SEM). The advantages of SEM are numerous and include: the sources of error across items comprising a latent variable are removed [22], the capacity to investigate relations between latent constructs as opposed to variables, and the ability to test comprehensive models that require subtle approaches to analysis of richly detailed specification [23]. The SEM was specified by transforming the measurement model, so that paths were introduced between the latent variables as indicated in Figure 1. All latent variables were defined by their respective items with associated error term. Latent variables regressed onto other latent variables (e.g., dentist anxiety from dentist reports of communication) required an associated ‘disturbance’ term according to conventional non-recursive constraints. No reciprocal paths were specified in accordance with Figure 1.

The first structural equation model was the overall model with all employed respondents included (n = 11,172) and second a set of SEMs, which simultaneously test the fit of the proposed model with the three major employment groups (managerial, intermediate and manual occupations). The parameter estimates were inspected closely between the latent variables labelled as communication, trust and dental anxiety. The hypothesised relationships between trust as communicated and dental anxiety, and communication by the dentist and dental anxiety were compared (a priori) between the three occupational groups as specified in the proposed model. An additional model was inspected with the simple modification of reversing the direction of the hypothesised influence from communication to dental anxiety. Constraints between the latent variables across the occupational groups were applied to formally test group variation. Conventional fit indices were employed to determine the closeness of the raw data conforming to the specified models, including the comparative fit index (CFI), Tucker-Lewis index (TLI), root mean square error of approximation (RMSEA) and chi-square. Chi-square values for assessing fit are not decisive by being oversensitive to large samples, however the CFI, TLI and RMSEA are instructive with suggested fit levels of ≥0.95, ≥0.95 and ≤0.05 that have been recommended were applied [24]. Large sample sizes produce precise parameter estimates (small confidence intervals) using maximum likelihood estimation [25].

Sensitivity analyses were run to confirm that the ordinal nature of the individual items was of little significance to the overall fit of the raw data to the specified model when data were promoted to interval level. Bayesian estimation was also performed to provide post-priori confidence intervals for reporting significance levels. The default overall convergence value for the posterior summaries was set to a conventional 1.002 [26]. Alpha (α) was set to 0.05. All *p*-values were two-sided. All analyses were performed by SPSS™ (IBM Corporation, Armonk, NY, USA) and AMOS v19™ [27].

## 3. Results

Of the 12,054 eligible households (HH), 7233 responded (60% HH response rate) the remaining 4821 HHs refused to participate. Within the 7233 HHs there were 13,509 adults who were invited to take part in the survey-of these 11,382 participated (84%). The HH response rate was 60% (7233 HHs) and an individual response rate (from within those HHs) of 84% (11,382 individuals) [14].

The sample size and summary data for the demographic variables across occupational status are presented in Table 2. The long-term unemployed and intermediate respondents were younger by an average of approximately 8 years of age from the remaining sample of 10,308 individuals. This group was removed from the SEM analysis when a detailed study of the model application across occupational status was made.

Objective 1: Estimation of the overall model fit

The fit of the measurement model with the total sample was excellent [(chi-square = 487.6, df = 118, CFI = 0.996, TLI = 0.995; RMSEA = 0.018 (95%CI: 0.016, 0.020)). Only 7 error covariances were introduced to correct minor distortion in the indicator specification of independent error terms. These were all theoretically trivial and considered unrevealing, hence requiring no adjustment of indicator membership to the assigned latent variables. No cross loadings were required. No negative variances or out of range values were detected. The factor loadings and correlations are presented in Table 1.

Objective 2: Determination of the relationship strength between patients’ trust, dentists’ communication, patient dental anxiety and shame

The structural equation model (SEM) including all three occupational groups demonstrated a high level of fit [(chi-square = 487.6, df = 118, CFI = 0.996, TLI = 0.995; RMSEA = 0.018 (95%CI: 0.016, 0.020)) and the standardised parameter estimates are presented in Table 3. The fit statistics were virtually identical to the measurement model. The key relationships showing the extent of association between Trust and Dental Anxiety, and Communication and Dental Anxiety were instructive and statistically significant. The second model reversing the direction of the path from communication to dental anxiety resulted in poorer fit (not shown) and this version was not studied further. Interpretation of the original hypothesised model showed consistent effects of shame about state of dentition, and past dental visiting behaviour associated with patients’ trust in the dentist and dental anxiety (standardized betas approximate to 0.2 regardless of valence sign: −ve or +ve). Dental anxiety was positively related to communication as defined by ‘having questions clearly answered’, ‘being given time to discuss’ and ‘being involved treatment discussions’ (stand. Beta = 0.20). Patients’ trust in the dentist, however, was significantly negatively related to dental anxiety (−0.31) and very strongly associated with communication (0.90).

Objective 3: Variation of the above relationships across social status

When the additional SEMs were fitted separating the sample into the three occupational groups, two patterns emerged. The paths between patients’ trust in the dentist and communication and the dependent variable dental anxiety varied in size according to social status. The manual SEP compared with the intermediate occupational SEP group showed strong relationships between patients’ trust in the dentist and dental anxiety; communication and dental anxiety (*p* < 0.05). No significant differences were shown in the strength of relationship between patients’ trust in the dentist and communication across the three groups. These effects were independent of sex as confirmed when separate analyses were conducted for men and women.

## 4. Discussion

Person-centred care has been emphasised and endorsed by the NHS with many positive advantages for patient health outcomes and improving healthcare service delivery [28]. As one of the underlying principles, effective communication is essential for delivering person-centred care [29]. The trusting relationship between the healthcare provider and the patient serves as a cornerstone for providing effective communication and therefore enabling a successful delivery of person-centred care. Patients’ psychosocial needs are a critical aspect of person-centred care, which is often expressed as dental anxiety. Feelings of shame due to poor oral health, as measured by patients’ self-consciousness and embarrassment about their dentition, may further exacerbate dental anxiety and fears. This requires dentists to provide emotional support to alleviate patients’ dental fears and anxiety. Prior research shows effective communication within the treatment alliance built on mutual trust can help reduce patient dental anxiety [30,31]. Although the relationship between high dental anxiety and delayed dental visiting behaviour has been well established [32], the mechanisms accounting for this association, including dentist communication, patient trust, dental anxiety and dental attendance has yet to be fully explored. The theoretical model proposed connects these person-centred factors with patient socioeconomic position (SEP) as a covariant to understand dental attendance behaviours. Our findings provide a good fit for our proposed theoretical model, however the authors fully acknowledge that due to the nature of SEM with the relatively large number of latent variables specified, that other models may be tested and provide an equally good or better fit.

Regarding the relationship strength between patient trust, dentist communication and patient dental anxiety and shame; patient trust, as hypothesised was negatively associated with dental anxiety [9,10,33]. More recently Jaakkola et al. showed a connection between high dental anxiety and reduced trust, together with increased shame and increased negative perceptions of dental health professionals [9,10,34].

Nevertheless, dentist communication was positively associated with dental anxiety, suggesting that patients given more time to discuss concerns and being involved as much as they wished, were more likely to be those who were dentally anxious. However, when these associations were investigated in greater depth across the occupation spectrum of the survey sample, a greater complexity emerged. It seemed that dentist communication to address patient dental anxiety, were weakly but significantly affected by patient SEP. Therefore, patients from professional/managerial and manual groups perceived the dentists’ communication as dental anxiety provoking while those in the intermediate group did not. How can such findings be explained?

Social rank theory would predict that those from lower socio-economic groups tend to withdraw from their interactions with health professionals as they would feel unable to use the health information provided [35]. Therefore, professional/managerial and/or intermediate classes would be expected to assimilate the dentists’ information while the opposite could be true for manual groups. Some evidence of poorer communication with low resourced patients (compared with affluent patients) from primary care health personnel has been shown especially with those patients with multiple health problems [36]. Social rank theory further suggests that there are inherent inequalities within society, which may result in shame, social anxiety and depression. For social rank theory, ‘shame, social anxiety and depression in small measure can be adaptive to the extent that they enable individuals to avoid serious social norm violations… [however when] they become maladaptive they set up viscous circles of increasing social avoidance and defensive submissive behaviours’ [37]. Therefore, we tentatively propose that the interaction between dentist communication and SEP is more complex and may give rise to poor social outcomes and exacerbating feelings of shame, loss and exclusion, regardless of SEP.

This speculative formulation has some bearing upon the individual who attends for dental treatment. We proposed that it is dental anxiety together with shame that reduces the establishment of a mutually trusting relationship or treatment alliance with the dental professional that acts as an intervening factor resulting in dental avoidance which has been shown in an infrequent pattern of dental attendance. This supposition is supported by work, which has investigated patient trust [9,10], patient shame [35] and dental anxiety [34] within the dentist-patient interaction. We suggest, therefore, that the inequalities in the dentist-patient interaction will be maintained by communication, which unintentionally promote patient anxiety and shame while reducing trust [37]. Effective dentist-patient communication has shown benefits of reducing patients’ dental anxiety and promoting their dental care experience [31]. In particular, communication skills such as active listening and showing empathy are useful to provide patients space to ventilate their anxiety and start to build the trusting relationship with their dentists. The finding that communication was associated with reduced dental anxiety and shame but increased trust tentatively supports the proposition that effective communication acts as a driver for regular dental visiting. Future work needs to concentrate on the improvement of self-reported measures of trust, communication and shame in order to examine these relationships, referred to here, in greater detail.

This report has benefited from a substantial sample size enabling complex testing of a number of associations and interactions simultaneously. Statistical fit levels were reassuring. Inevitably there are limitations. First, some compromises were made with scale construction as some of the items were drawn by the authors from question sets that were originally prepared by the ADHS investigators as general attitudinal content. This was especially the case with the communication and trust constructs which comprised from mixed items that were analysed singly without scale construction. Stronger psychometric scales of these two constructs would assist confirming the effects revealed in the model testing. Cross-sectional data can only partially reflect the possible causative models that have been hypothesised [38]. A longitudinal study would enable a more robust and testable confirmation of the causal links proposed. As with many structural equation models, there were multiple variations that could be applied. We adhered to those that made theoretical sense from our perspective. Nonetheless, this secondary analysis of the data proposes that communication is an important aspect of the dentist-patient interaction and a key element of person-centred care. Communicating with patients requires sophisticated and creative skills to ensure that anxiety and shame can be reduced, and trust maintained to enable those with high dental anxiety access dental care.

## 5. Conclusions

In conclusion, this report presents findings that suggests the potential importance of strengthening trust of all patients towards their dentist. This can be realised through effective dentist-patient interaction. Despite person-centred care being acknowledged widely as a core value for delivery of high quality dental care, there has been very limited research exploring this area [39]. The present work contributes some understandings of person-centred care using dental attendance as a lens to unravel the complex interplay between dentist’s communication, patient’s trust, shame and dental anxiety. The implications of these findings suggest that increased attention needs to be paid in future research to study the communication patterns between dentists and their patients, across the social gradient. Future work, therefore, should investigate more closely the interactions in dental settings, using video recordings and carefully coding of the interaction rather than using self-reported data [40].

## Figures and Tables

**Table 1:** Measurement model presenting indicator items and latent variable names with factor loadings and alpha coefficients.

Latent Variable Name	Item Name (Indicator)	No of Values (Bins)	Factor Loading	Alpha Coefficient
Dental anxiety	MDAS1 (dentist tomorrow)	5	0.94	0.92
	MDAS2 (waiting room)	5	0.97	
	MDAS3 (scale and polish)	5	0.80	
	MDAS4 (extraction)	5	0.67	
	MDAS5 (injection)	5	0.66	
Trust	Dentist listened carefully	2	0.73	0.75
	Dentist explained reasons for Rx	2	0.83	
	Was treated with respect	2	0.52	
	Had confidence and trust in dentist	2	0.62	
Communication	Got answers that could understand	3	0.67	0.78
	Was given enough time to discuss	3	0.77	
	Involved as much as wanted	3	0.75	
Shame	OHIP-14 self-conscious about teeth	5	0.83	0.84
	OHIP-14 embarrassed about teeth	5	0.88	
Past dental visiting	Regularity of attendance	4	0.91	0.93
behaviour	Frequency of visits in past	5	0.94	
(self-reported)	Last time when visited dentist	7	0.82	
	No. of visits in past 5 years	25	0.83	

**Table 2:** Socio-demographic characteristics of the study sample.

Occupational Group of Sample	N	%	Sex (% Female)	Age (Years)	
				**Mean**	**Sd**
Professional and managerial	3708	33.2	51	49.15	15.4
Intermediate	2289	20.5	61	50.86	16.0
Manual	4311	38.6	56	48.15	17.7
Never worked/Unemployed ^1^	864	7.7	60	40.70	22.6
Total	11,172	100	56	48.42	17.3

^1^ Includes not classified (n = 168).

**Table 3:** Standardized parameter estimates of model paths from the three occupational groups and combined sample including measures of fit.

		Professional and Managerial	Intermediate	Manual	Combined Sample
Key paths	Anxiety → Trust	−0.28	−0.17 ^a^	−0.45 ^a^	−0.31
	Commn → Anxiety	0.17	0.09 *^b^	0.35 ^b^	0.20
	Trust → Commn	0.88	0.87	0.92	0.90
Adjusted	Shame † →Anxiety	0.17	0.19	0.17	0.18
paths	Anxiety ‡ →Visiting	−0.20	−0.20	−0.25	−0.22
	Shame † →Trust	−0.19	−0.21	−0.20	−0.20
	Trust ‡ → Visiting	0.20	0.18	0.22	0.21
Model Fit	CFI	0.994	0.997	0.996	0.996
	TLI	0.992	0.997	0.994	0.995
	RMSEA	0.024	0.015	0.020	0.018
	Chi-square	338.2	173.7	285.1	487.6
	*p* level	<0.0001	0.001	<0.0001	<0.0001
N		3308	1976	3417	9520

All parameter estimates statistically significant (*p* < 0.01) with exception marked * (*p* < 0.05); ^a^ and ^b^: same superscript denotes statistically significantly different change in model fit between constrained and unconstrained parameters, thereby indicating substantive differences worthy of interpretation. ‡ high score describes frequent dental visiting. † high score describes high level of shame.
